# The efficient circulating immunoscore predicts prognosis of patients with advanced gastrointestinal cancer

**DOI:** 10.1186/s12957-022-02693-0

**Published:** 2022-07-12

**Authors:** Yamei Zhao, Yan Tang, Hanlin Qin, Kehai Feng, Changlu Hu

**Affiliations:** Department of Medical Oncology, The First Affiliated Hospital of USTC, Division of Life Sciences and Medicine, University of Science and Technology of China, Hefei, Anhui 230031 People’s Republic of China

**Keywords:** Gastrointestinal cancer, Circulating immune score, Circulating immune cells, Prognosis

## Abstract

**Background:**

Immunoscore from tumor tissues was initially established to evaluate the prognosis of solid tumor patients. However, the feasibility of circulating immune score (cIS) for the prognosis of advanced gastrointestinal cancers (AGC) has not been reported.

**Material and methods:**

Peripheral venous blood was collected from 64 untreated AGC patients. We utilized flow cytometry to determine several immune cell subpopulations, including CD8^+^ and CD4^+^ T cells, NK cells, and CD4 + CD25 + CD127^low^ Tregs. The circulating immune score 1 (cIS1) was assessed according to the proportions of CD4^+^, CD8^+^ T cells, and NK cell, whereas circulating immune score 2 (cIS2) was derived from the proportions of CD4^+^, CD8^+^ T cell, and CD4 + CD25 + CD127^low^ Tregs. The prognostic role of cIS for progression-free survival (PFS) and overall survival (OS) was analyzed using Kaplan–Meier curves and Cox multivariate models. Receiver operating characteristic (ROC) curves were depicted to compare the prognostic values of cIS1 and cIS2.

**Results:**

AGC patients with high cIS1(≥ 2) and cIS2(≥ 2) had significantly longer PFS (cIS1: median PFS, 11 vs. 6.7 months, *P* = 0.001; cIS2: 12 vs. 5.8 months, *P* < 0.0001) and OS (cIS1: median OS, 12 vs. 7.9 months, *P* = 0.0004; cIS2: 12.8 vs. 7.4 months, *P* < 0.0001) than those with low cIS1 and low cIS2. The areas under ROC curves (AUROCs) of cIS1 and cIS2 for OS were 0.526 (95% confidence interval; 95% CI 0.326–0.726) and 0.603 (95% CI 0.427–0.779, *P* = 0.332), whereas AUROC of cIS2 for PFS was larger than that of cIS1 0.735 (95% CI 0.609–0.837) vs 0.625 (95% CI 0.495–0.743) (*P* = 0.04)).

**Conclusion:**

The cIS can be applied to predict the prognosis of untreated AGC patients. Compared with cIS1, cIS2 displayed superior prognostic value for PFS prediction.

## Introduction

Gastrointestinal cancer (GC) is one of the most prevalent digestive tract tumors, and the leading cause of cancer-related morbidity and mortality worldwide [1]. Approximately 30–35% of gastric cancer patients presented with distant site metastases at the time of diagnosis [2], while 40–60% of colorectal cancer (CRC) patients have distant metastasis, of which 15–20% are simultaneous metastasis [3, 4]. Less than 25% of patients with advanced CRC are candidates for radical resection of liver and lung metastatic lesions [5]. However, those with peritoneal dissemination are less likely to undergo R0 resection or have a low survival rate [6, 7]. Patients with metastatic gastrointestinal tumors experience a high recurrence rate and low survival rate even if metastatic resection is feasible [8, 9]. Numerous traditional clinicopathological factors, including differentiated subtypes, lymph node condition of the primary tumor, distant metastatic sites, tumor burden, and feasibility of surgical resection, have been closely associated with patient survival in AGC. Various clinical scoring systems employ these indicators to predict patient prognosis [10, 11]. However, prognosis still varies even when patients receive the same treatment, demonstrating that these clinical indicators are not the only element affecting survival.

In recent years, tumor immune microenvironment has been critical in the occurrence and development of gastrointestinal tumors, tumor infiltration by T lymphocytes, including helper T cells (CD4^+^), cytotoxic T cells (CD8^+^), and T regulatory cells (Tregs, FOXp3^+^), is known to predict prognosis in multiple cancers including breast cancer, colorectal cancer, head and neck carcinoma, lung cancer, and esophageal cancer [12–20]. Immunoscore constructed by researchers, which considered the infiltration level of T cell subtype, B cell subtype or macrophage in tumor microenvironment (TME) of primary lesions or metastases, is widely applied to predict the prognosis in patients with gastrointestinal tumors [21–25]. In addition, more and more peripheral-related indicators are used to predict the prognosis of solid tumor patients [26–28]. The simplicity, reproducibility and accuracy of these scoring systems make them the most popular prediction models in clinical practice.

Considering that some patients with advanced tumors cannot acquire sufficient amounts of high-quality tissue specimens for quantitative analysis of tumor-associated immune cells and previous findings, we wondered whether the immune score could be extended to circulatory blood systems and identified it as a predictor of prognosis for unresectable AGC patients. Our research aimed to (1) construct a circulating immune score by determining the main subtypes of circulating immune cells and evaluating its prognostic effect on patients with untreated advanced gastrointestinal tumors and (2) compare the sensitivity and specificity of different circulating immune scoring systems for prognosis.

## Methods and materials

### Patient selection

This study included consecutive AGC patients who did not receive palliative treatment between July 2018 and April 2021 at the First Affiliated Hospital of University of Science and Technology in China. The inclusion criteria in this study are as follows: (1) pathologically diagnosed patients with gastric or colorectal carcinomas, and the existence of at least a distant metastatic site, (2) untreated AGC patients prior to diagnosis, (3) follow-up period greater than 6 months, and (4) existence of adequate pathological information and blood specimens for analysis. Exclusion criteria comprised those who (1) had autoimmune diseases or (2) were treated with long-term oral immunosuppressive therapy. The protocol for this research was approved by Institutional Review Board (IRB). All subjects provided written consent to participate in this investigation.

### Data collection

The patients were followed-up until November 2021 by medical records (inpatient and outpatient) or by telephone contact with patients or relatives aware of their illness. Clinical and pathological data were collected from electronic inpatient records. The data included pathological and histological type, distant metastatic site, expression of CEA and CA199 before the first treatment, ascites existence, physical status, etc. The expression proportion of circulating immune cell subtypes, including CD3^+^ T cells, CD4^+^ T cells, CD8^+^ T cells, NK cells, CD4^+^ CD25^+^ T cells, as well as the ratio of CD4^+^ CD25+ CD127^low^ regulatory T cells (Tregs), were specifically recorded.

### Flow cytometry

Peripheral blood samples were obtained before initiating therapy. Peripheral blood mononuclear cells (PBMCs) were splintered off from heparinized peripheral blood. Freshly isolated PBMCs or sorted cells (1 × 10^6^ cells) were stained with UCHT1-anti-CD3 (BioLegend, USA), RPA-T4-anti-CD4 (BioLegend, USA), HIT8a-anti-CD8 (Bio Legend, USA), MEM-188-anti-CD56 (BioLegend, USA), and APC-conjugated anti-CD127 (BioLegend, USA). The cells were then analyzed using a Navios flow cytometer (Beckman Coulter, USA) according to manufacturer’s instructions. The initial singlet gate was set on the lymphocytes on forward scatter and side scatter dot plots to depict immune cells to be analyzed in this study.

### Immune cell quantification

The best cutoff value of the expression proportion for each circulating immune cell subtype was selected by X-tile software V. 3.6.1. This software was used to measure the cutoff value of the expression proportions of CD3^+^ T cells, CD4^+^ T cells, CD8^+^ T cells, NK cells, CD4^+^ CD25^+^ T cells and CD4^+^ CD25^+^ CD127^low^ Tregs in patients, and for PFS, the best cutoff values were 58.1%, 29.5%, 19.3%, 17.7%, 14.6%, and 3.7%, respectively. For OS, the best cutoff points were 54.3%, 24.3%, 19.3%, 17.7%, 12.0%, and 4.0%, respectively. Each circulating immune cell subtype was given a dichotomous score (high expression levels of CD3^+^, CD4^+^, CD8^+^, NK cells scored 1, low expression level scored 0; low expression levels of CD4^+^ CD25^+^ CD127^low^ Tregs scored 1, high expression level scored 0) based on the set cutoff point. The circulating immune score 1 (cIS1) was based on expression levels of CD4^+^ T cells, CD8^+^ T cells, and NK cells. Patients with a cIS1 ≥ 2 points were classified as a high-score group (cIS1-high), whereas those with a cIS1 < 2 were classified as a low-score group (cIS1-low). Circulating immune score 2 (cIS2) was established according to expression levels of CD4^+^, CD8^+^, and CD4^+^ CD25^+^ CD127^low^ immune cells. Patients with cIS2 ≥ 2 points were classified as the high-score group (cIS2-high), whereas those with cIS2 < 2 were classified as the low-score group (cIS2-low).

### Statistical analysis and endpoint

The statistical analyses were executed using IBM SPSS software (version 22), GraphPad Prism (version 8.0.1), X-tile software (version 3.6.1), and MedCalc (version 19.0.4). The univariate and multivariate analyses were conducted using Cox proportional hazards model to select independent prognostic variables of prognosis. The optimal cutoff value for prognosis was determined based on X-tile software, and all patients were classified into high-level and low-level subgroups. The prognostic value of circulating immune score and various clinicopathological factors was analyzed using Kaplan-Meier curve and log-rank test. Cox multivariate models for PFS and OS were conducted for multivariate analysis to clear independent prognostic factors affecting survival of AGC patients. Receiver operating characteristic (ROC) curves were conducted to compare the predictive ability of cIS1 system with cIS2 system for PFS and OS. The primary endpoints of this study were OS and PFS. OS was defined as the time from the date of diagnosis to the date of death or last follow-up. PFS was defined as the time from the beginning of diagnosis to the date of diagnosis of treatment failure, death, or the last follow-up. *P* values < 0.05 using two-sided tests were considered statistically significant.

## Results

### Basic characteristics of AGC patients

This study retrospectively analyzed 64 untreated patients with an initial diagnosis of advanced gastrointestinal cancer. The average age of all patients was 62 years, including 18 (28.1%) females and 46 (71.9%) males. There were 36 cases (56.3%) of colorectal cancer and 28 cases (43.7%) of gastric cancer. A total of 14.1% of AGC patients were histologically diagnosed with mucinous adenocarcinoma or signet ring cell carcinoma. A total of 27 (42.2%) cases manifested with G1-2 histological grade, 31 (48.4%) cases with G3, whereas histological grade of 6 (9.4%) cases was unknown. At baseline, the imaging identified 48 (75%) patients with visceral metastasis and 33 (51.6%) patients with distant lymph node metastasis. The median expressing level of CD3^+^ T cells in peripheral blood was 68.5% (interquartile range, IQR, 60.65–76.28%), whereas that of CD8^+^ T cells was 28.85% (IQR, 20.28–34.23%). The median expressing level of CD4^+^ T cells was 37.15% (IQR, 28.65–41.18%), that of NK cells was 17.1% (IQR, 11.2–22.3%), while that of the ratio of CD4+CD25+CD127^low^ Tregs was 4.25% (IQR, 3.23–5.08%). These clinical characteristics are presented in Table 1.

**Table 1:** Clinical characteristics of patients with advanced gastrointestinal cancers

Variables	*N* (%)
Age, (mean)	62 (28–82)
Sex	
Female	18 (28.1)
Male	46 (71.9)
ECOG	
0–1	52 (81.3)
2	12 (18.7)
Primary cancer	
Colorectal cancer	36 (56.3)
Gastric cancer	28 (43.7)
Pathological type	
Non-adenocarcinoma/signet-ring cell cancer	55 (85.9)
Adenocarcinoma/Signet-ring cell cancer	9 (14.1)
Grade	
G1–2	27 (42.2)
G3	31 (48.4)
Unknown	6 (9.4)
CEA	
Abnormal	31 (48.4)
Normal	33 (51.6)
CA199	
Abnormal	24 (37.5)
Normal	40 (62.5)
Visceral metastasis	
Yes	48 (75.0)
No	16 (25.0)
Lymph node metastasis	
Yes	33 (51.6)
No	31 (48.4)
Disease progression	
Yes	31 (48.4)
No	33 (51.6)
Survival status	
Yes	10 (15.6)
No	54 (84.4)

### Prognostic role of different cIS in AGC patients

**Fig. 1:**
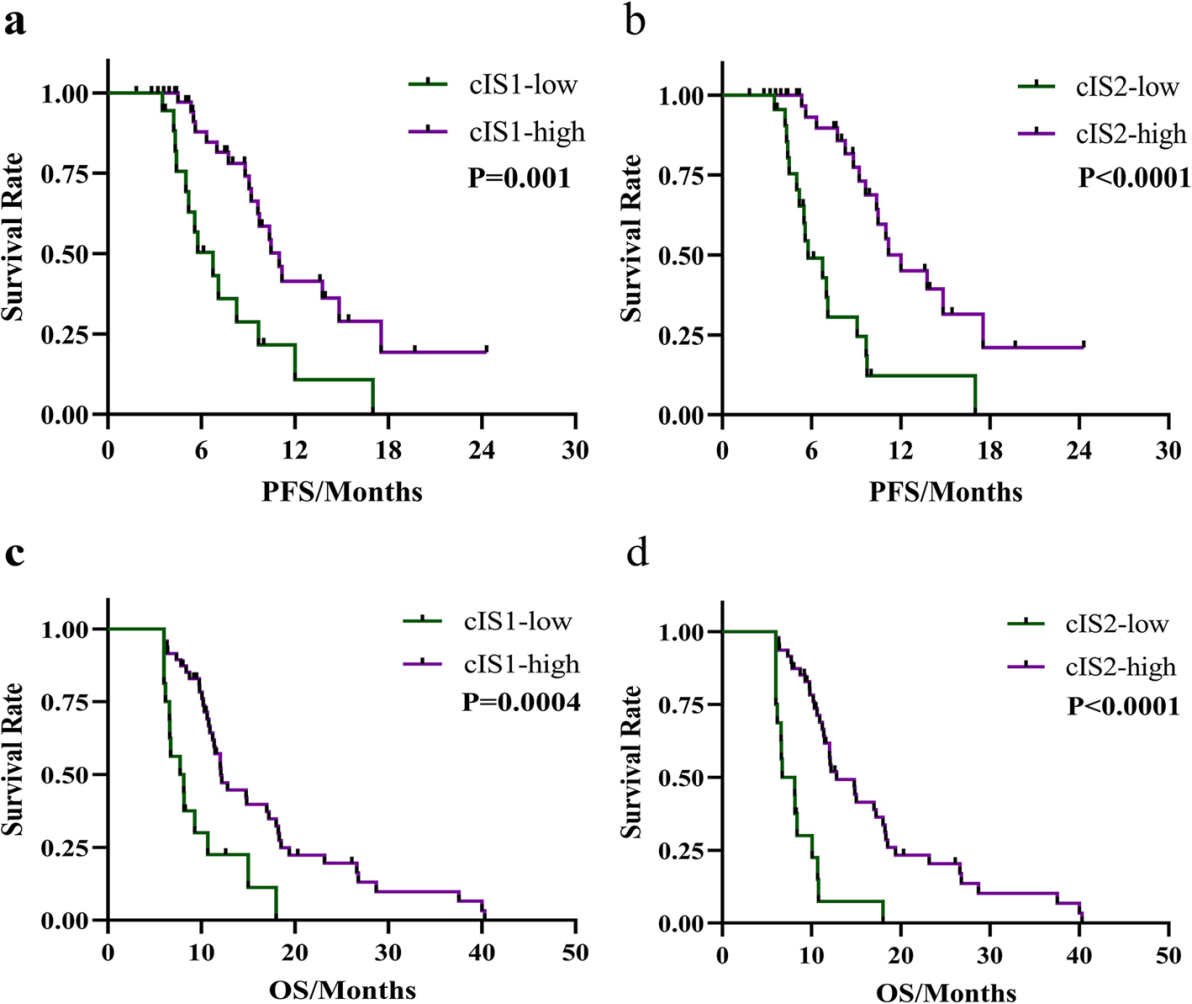
Kaplan-Meier estimates and log-rank test PFS according to cIS1 (**a**) and cIS2 score (**b**). Kaplan-Meier estimates and log-rank test OS according to cIS1 (**c**) and cIS2 score (**d**). **a**, **c** Patients with cIS1 ≥ 2 and cIS1 < 2 shown in purple and green, respectively. **b**, **d** Patients with cIS2 ≥ 2 and cIS2 < 2 displayed in purple and green, respectively. cIS = circulating immune score

Of 64 AGC patients who were followed up to November 2021, 48.4% had disease progression, while 15.6% died. The lost to follow-up rate was 3% (Two patients were lost to follow up). The median follow-up time was 11 months (IQR, 8.03–17.17 months). A total of 44 (68.7%) and 20 (31.3%) patients were categorized into high and low cIS1 subgroups, whereas 41(64.1%) and 23 (35.9%) were categorized into high and low cIS2 subgroups. The median PFS of cIS1-high and cIS1-low subgroups was 11 and 6.7 months, respectively (*P* = 0.001, Fig. 1a). The median PFS of cIS2-high and cIS2-low subgroups was 12 and 5.8 months, respectively (*P* < 0.0001, Fig. 1b). The difference in OS between high-cIS and low-cIS subgroups reach statistical significance (cIS1: median OS, 12 vs. 7.9 months, *P* = 0.0004 (Fig. 1c); cIS2: 12.8 vs. 7.4 months, *P* < 0.0001 (Fig. 1d)).

### Prognostic factors in univariate and multivariate analyses

For PFS, the results of univariate analysis of subtypes of circulating immune cells displayed that the expression level of CD4^+^ T cell (high vs. low, HR, 0.41, 95% CI (0.17–0.97), *P* = 0.01), CD8^+^ T cell (high vs. low, HR, 0.47, 95% CI (0.20–1.11), *P* = 0.03), CD4 + CD25 + CD127^low^ Treg (high vs. low, HR, 2.75, 95% CI (1.33–5.69), *P*= 0.027), cIS1 score (cIS1-high vs. cIS1-low, HR, 0.34, 95% CI (0.14–0.80) *P* = 0.001), and cIS2 score (cIS2-high vs. cIS2-low, HR, 0.26, 95% CI (0.11–0.59), *P* < 0.0001) had prognostic value. Other immune cell subtypes and clinicopathological factors were not significantly linked to PFS. In univariate analysis, it was found that the factors related to OS included the expression levels of CD8^+^ T cell (high vs. low, HR, 0.44, 95% CI (0.19–0.99), *P* = 0.006), CD4 + CD25+CD127^low^ Treg (high vs. low, HR, 1.87, 95% CI (1.10–3.20), *P* = 0.02), cIS1 score (cIS1-high vs. cIS1-low, HR, 0.36, 95% CI (0.16–0.84) *P* = 0.0004) and cIS2 score (cIS2-high vs. cIS2-low, HR, 0.26, 95% CI (0.10–0.66), *P* < 0.0001) (Table 2). After adjusting for clinicopathologic variables, multivariate Cox regression analyses revealed that both circulating immune scoring systems were independent predictors of PFS and OS (PFS: cIS1 score (cIS1-high vs. cIS1-low, HR, 0.41, 95% CI (0.19–0.86) *P* = 0.019), cIS2 score (cIS2-high vs. cIS2-low, HR, 0.16, 95% CI (0.06–0.4), *P* < 0.001). OS: cIS1 score (cIS1-high vs. cIS1-low, HR, 0.34, 95% CI (0.17–0.67) *P* = 0.002), cIS2 score (cIS2-high vs. cIS2-low, HR, 0.14, 95% CI (0.06–0.32), *P*<0.001) (Table 3).

**Table 2:** Univariate analysis for the prognosis of patients with advanced gastrointestinal cancers

Variables	PFS		OS	
	Univariate analysis		Univariate analysis	
	HR (95% CI)	*P* value	HR (95% CI)	*P* values
Age, years				
< 69 vs ≥ 69	0.53 (0.25–1.16)	*P = 0.11*	0.61 (0.31–1.20)	*P = 0.08*
Sex				
Female vs male	0.58 (0.28–1.21)	*P = 0.15*	0.76 (0.43–1.35)	*P = 0.36*
CEA				
Normal vs abnormal	0.98 (0.48–1.97)	*P = 0.95*	0.98 (0.57–1.67)	*P = 0.93*
CA199				
Normal vs abnormal	0.51 (0.25–1.01)	*P = 0.06*	0.63 (0.37–1.09)	*P = 0.41*
Grade				
G1–2 vs G3	0.76 (0.37–1.57)	*P = 0.47*	0.77 (0.43–1.36)	*P = 0.37*
Lymph node metastasis				
No vs yes	0.54 (0.27–1.10)	*P = 0.07*	0.81 (0.47–1.37)	*P = 0.40*
Visceral metastasis				
No vs yes	0.84 (0.38–1.86)	*P = 0.64*	0.96 (0.51–1.80)	*P = 0.90*
CD3 + cell				
High vs low	0.40 (0.13–1.27)	***P = 0.023***	0.36 (0.09–1.52)	***P = 0.02***
CD4 + cell				
High vs low	0.41 (0.17–0.97)	***P = 0.01***	0.40 (0.14–0.10)	*P = 0.07*
CD8 + cell				
High vs low	0.47 (0.20–1.11)	***P = 0.03***	0.44 (0.19–0.99)	***P = 0.006***
NK cell				
High vs low	1.92 (0.93–3.96)	*P = 0.053*	1.53 (0.86–2.70)	*P = 0.11*
CD4 + CD25+CD127^low^				
High vs low	2.75 (1.33–5.69)	***P = 0.027***	1.87 (1.10–3.20)	***P = 0.02***
cIS1				
High vs low	0.34 (0.14–0.80)	***P = 0.001***	0.36 (0.16–0.84)	***P = 0.0004***
cIS2				
High vs low	0.26 (0.11–0.59)	***P < 0.0001***	0.26 (0.10–0.66)	***P < 0.0001***

**Table 3:** Multivariate analyses of prognostic factors of patients with advanced gastrointestinal cancers

**OS**	**Multivariate analysis 1**		**Multivariate analysis 2**	
	HR (95% CI)	*P value*	HR (95% CI)	*P* value
Sex				
Female vs male	0.87 (0.46–1.63)	*P = 0.66*	0.75 (0.39–1.42)	*P = 0.37*
Age				
< 69 vs ≥ 69	0.78 (0.40–1.50)	*P = 0.45*	1.41 (0.65–3.06)	*P = 0.39*
Lymph node metastasis				
Yes vs no	1.42 (0.82–2.46)	*P = 0.22*	1.66 (0.93–2.95)	*P = 0.084*
cIS1				
High vs low	0.34 (0.17–0.67)	***P = 0.002***		
cIS2				
High vs low			0.14 (0.06–0.32)	***P < 0.001***
**PFS**	**Multivariate analysis 1**		**Multivariate analysis 2**	
Sex				
Female vs male	0.68 (0.28–1.65)	*P = 0.40*	0.51 (0.20–1.28)	*P = 0.15*
Age				
< 69 vs ≥ 69	0.68 (0.30–1.46)	*P = 0.31*	1.38 (0.52–3.66)	*P = 0.52*
Lymph node metastasis				
Yes vs no	2.03 (0.98–4.22)	*P = 0.056*	2.67 (1.26–5.68)	*P = 0.10*
cIS1				
High vs low	0.41 (0.19–0.86)	***P = 0.019***		
cIS2				
High vs low			0.16 (0.06–0.4)	***P < 0.001***

The bold value indicates *P* < 0.05, *HR* Hazard ratio, *CI* Confidence interval, sex, age, lymph node metastasis, and cIS1 were included in multivariate analysis 1; sex, age, lymph node metastasis and cIS2 were included in multivariate analysis 2

### Comparison of cIS1 and cIS2

**Fig. 2:**
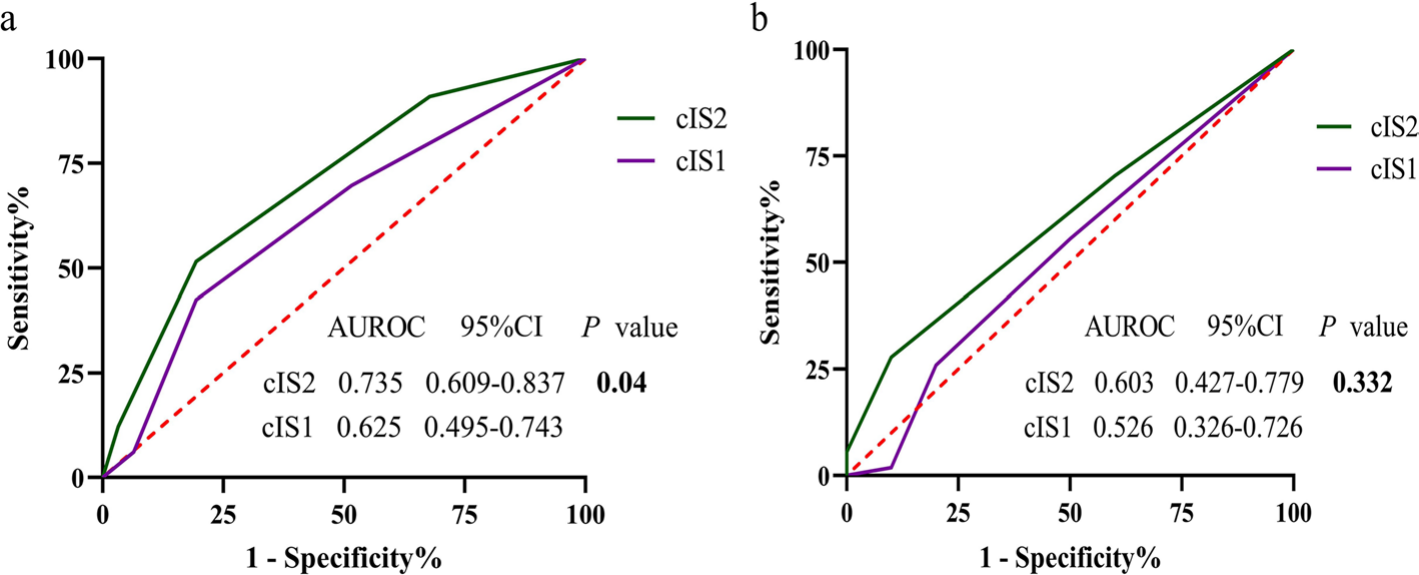
Comparison of the sensitivity and specificity for predicting PFS and OS of AGC patients with cIS1 and cIS2 systems. **a** ROC curves displayed the significant predictive values of both systems for PFS prediction, and cIS2 system was superior to cIS1 system in PFS prediction. **b** ROC curves indicated the difference between the two systems was insignificant for OS prediction. cIS = circulating immune score

ROC curves were performed to compare sensitivity and specificity of the predictive ability between cIS1 and cIS2 systems. The cIS2 corresponded to a relatively larger AUROC for PFS prediction than cIS1 system, with statistically significant difference (0.735 (95% CI 0.609–0.837) vs. 0.625 (95% CI 0.495–0.743) (*P* = 0.04)) (Fig. 2a). However, when OS prediction was compared, no statistically significant difference was observed between the two circulating immune scoring systems (*P* = 0.332, Fig. 2b).

## Discussion

In the current study, the circulating immune score system was first applied to assess the prognostic value in AGC patients. The results suggested that cIS system had the potential to predict the outcome of untreated AGC patients. This study successfully created a novel cIS1 scoring model based on an estimated total percentage of CD4^+^ T cells, CD8^+^ T cells, and NK cells in the peripheral blood, as well as a cIS2 scoring system based on the proportions of peripheral CD4^+^ T cells, CD8^+^ T cells, and CD4 + CD25 + CD127^low^ Tregs. Both scoring systems could serve as an effective index in predicting the outcome of AGC patients in either PFS or OS; high cIS was significantly associated with extended OS and PFS in the survival analysis.

For patients with resectable gastrointestinal cancer, various studies have demonstrated that individual tumor-infiltrating lymphocyte subtypes and the ratio of subsets in tumor microenvironment (TME) are critical predictors of survival and recurrence [13, 18, 21, 29–31]. In recent years, accumulating studies have employed TIL to create an immune scoring system to predict the prognosis of patients with non-advanced gastric cancer or colorectal cancer. Meanwhile, some researchers have investigated the infiltration of tumor-associated immune cells in the metastatic foci of gastrointestinal tumors to establish a novel immune scoring system to analyze its prognostic role in metastatic gastrointestinal tumors. Pagès et al. stated that patients with stage I–III colon cancer with a high immunoscore were associated with the lowest risk of 5-year recurrence [23]. Similarly, Jiang et al. revealed that for patients with stage II-III gastric cancer, compared with the low immunoscore group, the high immunoscore predicted higher 5-year disease-free survival (45.0% vs. 4.4%, respectively; *P* < 0.001) and 5-year overall survival (48.8% vs. 6.7%, respectively; *P* < 0.001) [32]. IS and TB scoring systems of metastatic focus constructed by Wang et al. and Mlecnik et al. using immune cell subtypes after resection of common metastatic sites of colorectal cancer (liver, lung, peritoneum, etc.) also revealed good independent predictive values [22, 24]. At present, increasing studies on the exploratory analysis of immune cell subtypes in peripheral blood, regarding its prognostic value, indicated that expression levels of circulating regulatory T cells, NK cells, CD3^+^ T cells, CD4^+^ T cells, and CD8^+^ T cells were also significantly linked to survival [33–36]. Although the above immune score concerning TME demonstrates good predictive function, its evaluation process remains very complex, limiting its extensive application in clinic.

This study performed immune scoring according to peripheral blood immune cell subtypes of AGC patients, which was applied to patients with initially resectable or initially unresectable advanced tumors. Although previous studies involving the immunoscore of TME have confirmed that it could be used as a supplement to TNM staging, and even the prediction efficiency was better than TNM staging, the evaluation criteria for immune cells of each study were different. These researches required sufficient tissue samples and professional pathologists to distinguish types, density, functional localization, and location of adaptive immune cells in different tumor regions [23, 32]. For AGC patients under heavy tumor burden with the inability to obtain enough tumor tissue samples due to loss of operation opportunity, the immune score for TME could not be evaluated. Our current study expanded the immunoscore from TME to peripheral blood and preliminarily explored the prognostic prediction of initial untreated AGC using the circulating immune score constructed by peripheral blood immune cells subtypes. The preliminary results indicated that cIS had a certain predictive value and was straightforward and feasible; it was not limited to whether patients with tumors could obtain sufficient tumor tissue specimens.

CD8^+^ T and CD3^+^ T cells were significant subtypes of tumor-related immune cells; the former played an anti-tumor role directly or indirectly through cytotoxicity, and the latter activated and promoted proliferation and acted as effector molecules of CD8^+^ T and NK cells. In solid tumors, the high-density infiltration of these cells was strongly linked to a favorable prognosis [13, 18, 37, 38]. Tumor tissue-derived and peripheral blood-derived NK cells are damaged in vivo in cancer patients. On the one hand, it might be due to depletion and functional destruction of functional NK cells. On the other hand, it might be due to the existence of immunosuppressive targets (such as PD-L1) in malignant tumor patients, resulting in the damage of NK cell-induced ADCC cytotoxicity. In previous studies, such damage indicated tumor progression and poor prognosis [39–41]. Pernot et al. revealed that a high count of circulating NK correlates with a better prognosis in gastric cancer [36]. CD4+ CD25+ CD127low/-T cell population had the most typical Treg characteristics. CD127 with low or no expression on CD4+ CD25+ markers could recognize Treg more accurately than the combination of other markers [42]. Treg cells promote immunosuppression by inhibiting immune response to cancer cells, and the expression of CD4+ CD25+ CD127^low^ Tregs had a positive correlation with TGF-β1 and IL-10 expressions and was closely linked to tumor occurrence and development and immunotherapy reactivity [33, 43]. Our study indicated that individual circulating tumor-related immune cells also demonstrate prognostic value similar to previous studies. In addition, the scoring system constructed by combining multiple circulating immune cells for the first time displayed good performance in predicting clinical outcomes. Moreover, this study revealed that the prediction performance of cIS2 based on CD4^+^ T cells, CD8^+^ T cells, and Treg cells outperforms that of cIS1 based on CD4^+^ T cells, CD8^+^ T cells, and NK cells, which also provides a certain direction for our future research on peripheral blood immune score.

This study displayed the first use of circulating immune score based on multiple immune cell subtypes in peripheral blood to predict prognosis in AGC patients. This study included patients with gastrointestinal tumors and simultaneous metastasis and was untreated initially, avoiding the impact of systematic treatment on the patient's immune system before collecting blood samples. However, this research was designed as a preliminary exploratory study and lacked independent validation set for external or internal validation of the immune score, which crippled a certain degree of credibility. In addition, current results were limited by small sample size, single-center, and retrospective design. Inconsistencies in the follow-up treatment of selected patients, including palliative surgery or palliative chemoradiotherapy or immunotherapy, or participation in clinical trials organized by our center, would also cause inevitable confounding factors. As a result, additional investigations involving expanding the sample size and the number of research centers are necessary.

## Conclusion

In conclusion, this study demonstrated that a circulating immune score system could be deployed to predict the prognosis of patients with initial untreated AGC. We confirmed that cIS1 system constructed by combining the expression proportions of CD4^+^, CD8^+^ T cells, and NK cells, as well as cIS2 system constructed by combining CD3^+^ T cells, CD8^+^ T cells, and CD4+ CD25+ CD127low Tregs, could successfully predict PFS and OS of AGC patients. Furthermore, cIS2 displayed a superior prognostic value to cIS1 in PFS prediction.

## Data Availability

All data generated or analyzed during this study are included in this published article.

## Abbreviations

cIS: Circulating immune score
AGC: Advanced gastrointestinal cancers
OS: Overall survival
PFS: Progression-free survival
GC: Gastrointestinal cancer
TME: Tumor microenvironment
PBMCs: Peripheral blood mononuclear cells
